# Epidemiology of acute infections in people who inject drugs in Australia

**DOI:** 10.1111/dar.13772

**Published:** 2023-11-23

**Authors:** Lucy O. Attwood, Daniel O'Keefe, Peter Higgs, Olga Vujovic, Joseph S. Doyle, Andrew J. Stewardson

**Affiliations:** ^1^ Department of Infectious Diseases, The Alfred Hospital and Central Clinical School Monash University Melbourne Australia; ^2^ Burnet Institute Melbourne Australia; ^3^ Department of Public Health La Trobe University Melbourne Australia

**Keywords:** epidemiology, health systems, injecting drug use, sepsis, skin and soft tissue infections

## Abstract

**Issues:**

People who inject drugs are at risk of acute infections, such as skin and soft tissue infections, infective endocarditis, bone and joint infections and bloodstream infections. There has been an increase in these infections in people who inject drugs internationally over the past 10 years. However, the local data regarding acute infections in Australia has not been well described.

**Approach:**

We review the epidemiology of acute infections and associated morbidity and mortality amongst people who inject drugs in Australia. We summarise risk factors for these infections, including the concurrent social and psychological determinants of health.

**Key Findings:**

The proportion of people who report having injected drugs in the prior 12 months in Australia has decreased over the past 18 years. However, there has been an increase in the burden of acute infections in this population. This increase is driven largely by skin and soft tissue infections. People who inject drugs often have multiple conflicting priorities that can delay engagement in care.

**Implications:**

Acute infections in people who inject drugs are associated with significant morbidity and mortality. Acute infections contribute to significant bed days, surgical requirements and health‐care costs in Australia. The increase in these infections is likely due to a complex interplay of microbiological, individual, social and environmental factors.

**Conclusion:**

Acute infections in people who inject drugs in Australia represent a significant burden to both patients and health‐care systems. Flexible health‐care models, such as low‐threshold wound clinics, would help directly target, and address early interventions, for these infections.

**Key Points**

- This paper summarises the current evidence regarding the burden of and risk factors for acute infections amongst people who inject drugs in Australia.
- Acute infections are increasing, driven primarily by skin and soft tissue infections in Australia.
- People who inject drugs often manage multiple comorbidities and barriers to care that may delay treatment for these acute infections.
- Acute infections frequently require prolonged hospital admissions and a significant cost to the Australian health‐care system.
- Changing patterns of antimicrobial resistance, an increase in the use of crystal methamphetamine and an ageing population of people who inject drugs may all be contributing to the increase in acute infections.

## 1 INTRODUCTION

There has been an increase in acute bacterial and fungal infections in people who inject drugs reported in the past 10 years globally including in the United States [1, 2, 3, 4, 5], Canada [6, 7], South Africa [8] and India [9]. These infections can range from local skin and soft tissue infections (SSTI) to invasive infections such as infective endocarditis (IE), bone and joint infections and bloodstream infections. The term ‘acute infections’ will be used in this paper to refer to these bacterial and fungal infections [10], though we acknowledge that people who inject drugs are at risk of acute blood borne viruses such as hepatitis C. Acute infections are a significant cause of morbidity and mortality amongst people who inject drugs. Furthermore, people who inject drugs hospitalised with acute infections often have longer hospital admissions and more frequent unplanned readmissions than non‐injecting drug use related hospitalisations for the same infections [3, 11, 12, 13].

A local understanding of the epidemiology of acute infections amongst people who inject drugs is necessary to develop patient‐centred care that reflects the current needs in Australia. This review will summarise the current data regarding the epidemiology of acute infections in people who inject drugs in Australia. We review the frequency, risk factors and outcomes of these infections, discuss the social determinants of health that may be contributing to the current increase in infections, and highlight the limitations of currently available data.

## 2 METHODS

We searched Ovid MEDLINE and EMBASE, PubMed and Google Scholar through to July 2023 for clinical trials, cohort studies, case series and reviews related to acute infections in people who inject drugs. Examples of search terms used include ‘injection drug use’, ‘people who inject drugs’, ‘endocarditis’, ‘osteomyelitis’, ‘skin and soft tissue infection’, ‘bacterial infection’ and ‘epidemiology’. We also manually searched the reference lists of identified articles for other relevant articles. Data from Australia were prioritised, with key studies from other countries included for context.

## 3 FREQUENCY OF ACUTE INFECTIONS IN AUSTRALIA

Acute infections in people who inject drugs are common, with a 2017 systematic review finding up to one‐third of people who inject drugs had experienced injection site infections within the past month [14]. Invasive infections including IE and sepsis were experienced by up to 10% in their lifetime [14]. There are widely varying estimates of the lifetime prevalence of acute infections amongst people who inject drugs, in part explained by methodological differences between studies. SSTI is the most frequent, reported as 6–69% [12, 13, 14, 15]. This is followed by sepsis (2–10%), IE (0.5–12%) and bone and joint infections (0.5–2%) [12, 13, 14, 15].

There is no gold‐standard method to determine the population size of people who inject drugs, which is an important barrier to estimating the rate of acute infections in people who inject drugs [16]. Larney et al. estimated the number of people who inject drugs in Australia to be between 68,000 and 118,000 in 2017, though noted that there are limited data available to inform estimates [17]. Based on the 2019 Australian National Drug Strategy Household Survey, the proportion of people who have injected drugs in the past 12 months in Australia declined from 0.6% in 2001 to 0.3% in 2019 [18]. There has also been a decline in both hepatitis C incidence and prevalence across Australia following the availability of unrestricted direct acting antiviral therapy in March 2016 [19, 20, 21]. Improved harm reduction measures such as opioid agonist treatment uptake and needle and syringe programs have also been credited with the reduction [19, 20, 21, 22].

The improvements seen in the incidence and prevalence of hepatitis C do not appear to be reflected in the frequency of acute infections in people who inject drugs in Australia. Available data indicates that acute infections amongst people who inject drugs are common in Australia. The 2021 Illicit Drug Reporting (IDRS) Australian survey found that 26% of participants had experienced an injection‐related health issue in the previous month, of which 8% involved infection [23]. Reflecting international data, SSTI are the most common acute infections in Australia with self‐reported lifetime prevalence of 6–23% [24, 25, 26]. This is much higher than other acute infections with the lifetime self‐reported prevalence of septicaemia 2%, endocarditis 1% and bone and joint infections <1% [24, 25].

Acute infections amongst people who inject drugs represent a significant burden on the Australian health‐care system. A cohort study in New South Wales of 8943 participants between 2001 and 2018 found that SSTI (79%, *n* = 7021) were the most common infectious cause of index hospitalisation [27]. Further, a recent Australian study reported that of 1851 participants, one in five (20%, 377) had been admitted to hospital in the past year for a SSTI [28]. This was reflected in an Australian longitudinal study of opioid agonist therapy participants, which estimated the incidence of SSTI‐related hospital separations at 29.9 per 1000 person‐years, with 15% of the cohort (6973 individuals out of the 47,163 total cohort) requiring hospitalisation for a SSTI during the study period (August 2001–December 2017) [29]. A retrospective review of acute infections amongst people who inject drugs at a tertiary hospital in Melbourne between January 2017 and April 2019 mirrored this finding, reporting that SSTI was the most common infection (119/205, 58%), followed by bacteraemia (36/205, 18%) and endocarditis (26/205, 13%) [30].

Of concern, the New South Wales cohort study found that systemic infections (rather than SSTI) represented a higher proportion of hospitalisations than documented in the previous self‐reported studies, with sepsis/bacteraemia documented in 14% (1207/7021) and endocarditis in 5% (431/7021) [27]. These differences may be due to some of the inherent limitations of self‐reporting studies. Injecting drug use is an illegal behaviour with a highly marginalised population. Thus, people may be hesitant to openly disclose information regarding their injecting drug use and associated complications. As a result, population surveys risk underestimating both the prevalence of injecting drug use and related harm [31, 32]. Self‐reporting surveys also depend on participant recall and health literacy. While participants may be able to accurately describe symptoms suffered, they may not have sought, or be able to provide, an accurate diagnosis, limiting the precision of clinical data. Recent studies from New South Wales presented detailed analysis of the current burden of acute infections through data‐linkage of participants accessing opioid agonist therapy (OAT), providing a different source of this evidence base [27, 29]. These studies were both retrospective and it should be noted that a prospective multicentre cohort study is currently enrolling in Australia to try and determine the current epidemiology of acute infections in people who inject drugs [33].

There is evidence that acute infections amongst people who inject drugs in Australia is increasing. Colledge‐Frisby et al. found that amongst participants accessing OAT in New South Wales between 2001 and 2017, the age‐adjusted incidence rate of hospitalisation for injecting‐related diseases increased from 34.8 to 54.9 per 1000 person years [29]. A recent Melbourne analysis at a tertiary hospital using International Classification of Diseases codes found that over 12 years between 2008 and 2020, acute infections increased from 138 to 249 per 100,000 admissions [34]. The burden of admissions was driven by SSTI, which accounted for nearly half of all admissions for people who inject drugs with acute infections (797/1751 = 46%) [34]. The majority of SSTI (86.5%, 689/797) were uncomplicated [34] and thus indicate a potential opportunity for improved early intervention and treatment to prevent hospitalisation. Globally, there has been a dramatic rise in the cases of IE related to injecting drug use in the past 10 years, and it is estimated that people who inject drugs now comprise 8–38% of all cases of IE [35, 36, 37]. This trend has also been documented in Australia with the incidence of injecting drug use‐related IE increasing from 0.9 to 1.8 per 100,000 people per year between 2009 and 2014 based on data from the Victorian Admitted Episode Dataset [38]. Infective endocarditis in people who inject drugs had a rate ratio of 1.9 (95% confidence interval [CI] 1.3–2.9), higher than the rate ratio of IE overall, which was 1.2 (95% CI 1.1–1.4) [38].

## 4 RISK FACTORS FOR ACUTE INFECTIONS IN PEOPLE WHO INJECT DRUGS

**FIGURE 1:**
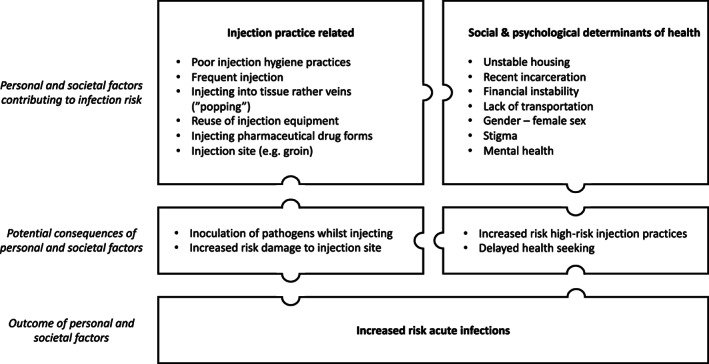
Intersecting factors contributing to the occurrence of acute infections in people who inject drugs.

Injecting drug use can directly increase risk of acute infections through the inoculation of pathogens while injecting. The risk of infection is increased through inadequate hygiene practices, frequent injecting, injecting into tissues rather than veins (‘popping’) as well as the reuse of injection equipment which may result in blunt needles, increasing the risk of damage to the injection site [10, 14, 39, 40, 41, 42] (Figure 1). In Australia, the reuse of needles is of particular concern, with a 2019 survey of 897 people who inject drugs as part of the IDRS finding that nearly half of participants (43%, 389/897) reported re‐using their own unsterile needle/syringe [26]. There is also ongoing receptive sharing of needles in Australia. The 2021 Australian Needle Syringe Program survey found that 18% of respondents reported receptive sharing of needles and syringes (injecting with another person's unsterile needle/syringe) in the last month [21]. A systematic review and meta‐analysis found that binge drug use and methamphetamine use were positively associated with the receptive sharing of used syringes [43]. This is of particular relevance in Australia as the 2021 IDRS demonstrated that methamphetamine has now surpassed heroin as the most common drug of choice in Australia [23]. The Australian Needle Syringe Program Survey also noted an increase from 41% to 51% of respondents reporting methamphetamine injection between 2017 and 2021 [21].

Amongst people who inject drugs, those of female sex, people recently released from prison and people with unstable housing have an increased risk of SSTI [10, 14, 28]. These groups have an increased risk of engaging in high‐risk behaviours including public injecting and equipment sharing, which limit the ability to maintain hygienic injecting practices [28]. Other practices that have been found to be associated with acute infections include injecting pharmaceutical drug forms (such as tablets and gel capsules) and in high‐risk injecting sites, such as the groin [40, 42, 44]. Pharmaceutical medications may predispose individuals to increased risk of acute infections, particularly SSTI, as tablets must be crushed and dissolved before they can be injected, increasing the risk of bacterial or fungal contamination [45]. Injecting pharmaceutical drugs has also been associated with an increased risk of vascular damage, especially if injected without filtration to remove large particulates [42, 46]. There has been an increase in injection of pharmaceutical opioids across the world, including in Australia [45]. The 2021 Needle Syringe Program Survey demonstrated that the third most commonly reported class of drugs last injected nationally (after methamphetamine and heroin) was pharmaceutical opioids (including morphine, oxycodone and fentanyl) [21].

There are many social, structural and psychological factors that impact on the risk of people who inject drugs developing acute infections and seeking timely health care for these infections. These include injecting drug use related stigma, unstable housing, significant mental health burden and a lack of continuity to care experienced by many people who inject drugs (Figure 1). People who inject drugs experience a higher rate of mental illness than the general public. A systematic review found that amongst people who inject drugs, the pooled estimate of current severe depressive symptomology was 42% (95% CI 21.3%–62.8%) and 28.7% (95% CI 20.8%–26.6%) had a diagnosis of depression [47]. This is much higher than the 21% of Australians aged 16‐85 years estimated to have experienced a mental disorder, with 8.8% of the population reporting a long term mental health condition  [48, 49]. This high burden amongst people who inject drugs was reflected in an analysis of injecting injuries and diseases in Australia, with 402 out of 897 (45%) reporting mental health issues in the previous 6 months [26]. This percentage increased to 73% (38 out of 52) when the high burden group of injecting injuries and diseases was analysed [26]. Psychological distress has been linked to increased risk of binge injecting drug use and sharing of injecting equipment, increasing the risk of harm from injecting drug use [50].

Mental health comorbidities can also be exacerbated by the significant social stressors commonly experienced by people who inject drugs. There are often competing priorities including housing instability, financial pressures, lack of transport and legal undertakings that may contribute to delayed presentation to care for acute infections [28, 51, 52]. A retrospective review of acute infections at a tertiary hospital in Melbourne between January 2017 and April 2019 found that nearly half (49%, 111/226) had unstable housing and over 50% (96/178) had a psychiatric diagnosis [30]. This was also reflected in an analysis of emergency department presentations of people who inject drugs in Melbourne, Victoria, with nearly a fifth of presentations reporting homelessness [53]. People who inject drugs may defer seeking healthcare due to concerns about injecting drug use related stigma, which in turn affects their ability to seek health care [54]. An Australian cohort of people who inject drugs reported that 24% experienced discrimination monthly, 16% experienced discrimination weekly and 13% experienced discrimination daily or more [55]. Stigma can result in delayed presentation and hesitancy to present for acute health care, lack of trust in health‐care providers, a reliance on self‐care and higher rates of unplanned discharges from hospital [56, 57, 58, 59, 60, 61, 62, 63]. Stigma and marginalisation can also reduce engagement with harm reduction services, increasing the possibility of high‐risk injecting techniques [26, 64].

## 5 FACTORS CONTRIBUTING TO THE INCREASE IN ACUTE INFECTIONS IN PEOPLE WHO INJECT DRUGS IN AUSTRALIA

The increase in acute infections in people who inject drugs in Australia has occurred despite a decrease in the estimated population size of people who inject drugs in Australia. This change in epidemiology is likely due to an interplay of microbiological, individual, social and environmental factors.

Amongst people who inject drugs, changes in clones of bacteria and the prevalence of resistant organisms such as methicillin‐resistant *Staphylococcus aureus* (MRSA) are of increasing concern. Clonal expansion of *S. aureus* and *Streptococcus pyogenes* have contributed to outbreaks of acute infections in people who inject drugs in the United Kingdom and Europe [65, 66, 67]. Furthermore, acute infections due to MRSA have been increasingly reported in people who inject drugs in North America and Europe, with concerns around shifts in population structures of MRSA clones [66, 68, 69]. While *S. aureus* is a priority organism for antimicrobial resistance research in Australia [70], there is very little information about the molecular epidemiology of MRSA amongst people who inject drugs in Australia. This is clearly an area where future research is needed.

The rising incidence of acute infections may also be related to the increased use of methamphetamine in Australia [23]. Eighty‐one percent of people interviewed as part of the IDRS interviews in 2022 reported methamphetamine use within the past 6 months, compared to 60% in 2010 [23]. Methamphetamine use is associated with high‐risk behaviours and drug‐related harms and there is clear evidence that hospital admissions related to crystal methamphetamine use have been increasing in Australia since 2010 [43, 71, 72].

People who inject drugs in Australia are an ageing cohort [21, 23]. While older people who inject drugs have been found to report lower high‐risk injecting behaviours [73], a longer duration of injecting may increase the risk of vascular damage and access issues, increasing the risk of acute infections, especially SSTI [40, 74]. Furthermore, the number of people experiencing homelessness at the time of the 2021 Australian census increased by 5% compared to the 2016 census [75]. Unstable housing limits the ability to practice safe injecting and thus may also be contributing to the increase in acute infections in Australia [76, 77].

It is also important to acknowledge that the COVID‐19 pandemic has impacted people who inject drugs in Australia in many ways. While the two supervised injecting facilities in Australia remained open [78], many secondary programs (including needle and syringe programs) were closed or had altered hours, with resultant disruptions in access to harm reduction and drug treatment services [79, 80]. Medical appointments including OAT provision often transitioned to telehealth, removing an opportunity to screen for SSTI and acute infections [79]. However, some interventions, such as providing temporary accommodation to those with unstable housing, may have decreased rates of acute infections. A 35% reduction in the rate of acute infections was found in England during COVID‐19, hypothesised to be due to the reduction of social mixing and access to hygienic environments through the hotel program provided during the pandemic [81]. Further research is required in Australia to determine the impact of the pandemic on acute infections and injecting‐related behaviours.

## 6 IMPACT OF ACUTE INFECTIONS IN PEOPLE WHO INJECT DRUGS IN AUSTRALIA

Acute infections are associated with significant morbidity to patients. An analysis of admissions by people who inject drugs to a tertiary hospital in Melbourne with acute infections found that one‐third of admissions required surgery (77/226) [30]. Length of stay for systemic infections was over 2 weeks, with a mean of 15 days (interquartile range 9–38) [30]. The receipt of care was also complicated, with nearly one‐third of patients experiencing an unplanned discharge (66/226, 30%) [30]. Patient‐directed discharges are associated with poor outcomes including high readmission rates and increased mortality [82]. A high rate of incomplete treatment was also documented in an Australian analysis of people who inject drugs with *S. aureus* bacteraemia, with people who inject drugs nearly five times more likely to experience incomplete treatment compared to people without a history of injecting drug use [83].

In general, hospitalisations of people who inject drugs with infectious complications often result in longer hospital stays, higher readmission rates and higher hospital charges, than non‐injecting drug use related hospitalisations for the same diagnoses [3, 12, 13, 84, 85, 86, 87, 88]. Compared to other cases of endocarditis, IE resulting from injecting drug use is associated with 70% longer length of stay, and nearly twice the costs for the health‐care service [89]. Even when an acute infection is not systemic, people who inject drugs with SSTIs tend to have longer hospital stays (median 4 days) compared to other hospitalised patients (median 2 days) [90]. This may be driven by more severe presentations. An analysis of IE in people who inject drugs at a Melbourne tertiary hospital found a significant disease burden, with the majority of patients exhibiting evidence of emboli (*n* = 40, 73%) and a large vegetation ≥1 cm (*n* = 52, 58%) [91]. Total mortality was high at 14.5%, with surgical mortality 10% [91]. A separate Australian retrospective study of injecting drug use related IE between 1997 and 2015 documented a long median inpatient stay of 37 days (interquartile range 1–84) and severe clinical presentations, with 56% of episodes requiring an intensive care unit admission (71/127) [44]. This compares to a previous Australian study of all IE admissions in New South Wales between 2000 and 2006, in which 24% of the cohort required intensive care unit admission [92]. These severe presentations occur even though people who inject drugs are usually younger with fewer comorbidities than patients with no injecting drug use history, and outcomes for IE are similar between these two groups for this serious disease [93].

When considering acute infections in people who inject drugs, there is a higher risk of rehospitalisation than in patients without a history of injecting drug use. Low et al. found a high burden of repeat episodes of IE in people who inject drugs in Victoria, occurring in 34/127 (27%) [44]. This compares to published rates of recurrence of IE in the general population of between 2% and 6% [94]. No significant difference has been found in in‐hospital mortality nor 30‐day mortality between people who inject drugs and people with no drug use history following valve surgery for IE [95]. However, poorer outcomes have been documented amongst people who inject drugs for mid‐ and long‐term outcomes post‐surgery internationally impacted by increased risk of reinfection [96, 97, 98]. Brothers et al. found that 41% (3653/8943) of participants were rehospitalised during the study period (1 July 2001 to 28 June 2018) with an acute infection [27]. The majority of rehospitalisations were for SSTI (78%, *n* = 2718), with bloodstream infections being documented in 15% (*n* = 556) [27].

The severe presentations, frequent rehospitalisations and prolonged admissions amongst people who inject drugs with acute infections contribute a significant cost to the Australian healthcare system. The average cost per episode of IE at a Victorian tertiary centre was over $AU74,000 [44]. A separate study out of Victoria found the total medical cost for 22 episodes of IE related to injecting drug use was $1.6 million [99]. This high cost is also seen in a recent Australian review of spinal infections, where the average expenditure per episode was $AU61,577 [100]. It has been estimated hospitalisation costs for acute infections are as much as the costs of treatment in the community, despite resulting from far fewer episodes of care [101]. The cost to the public health system over a 12‐month period in 2005 and 2006 of treating non‐viral injuries and diseases was estimated to be $AU20 million in Queensland, New South Wales and Victoria [101]. This included between 8496 and 14,044 hospital bed days in the public hospitals of those states [101].

## 7 IMPLICATIONS AND FUTURE RESEARCH

Available data demonstrates that the frequency of acute infections in people who inject drugs in Australia is increasing. This is despite a stable population of people who inject drugs, good coverage of needle and syringe programs and reductions in hepatitis C incidence and prevalence [18, 19, 21]. Strategies that directly target and address early intervention for acute infections are required to prevent the ongoing increase in infections. Low‐threshold wound clinics based within services already accessed by people who inject drugs, such as needle‐syringe programs, supervised injecting facilities and OAT providers, would allow early access to health care and prompt medical treatment. This in turn could help prevent escalation of SSTI to systemic infections requiring hospitalisation. Providing wrap around services, including social and psychological support, could also help address some of the factors that predispose to acute infections. A flexible, drop‐in skin and soft tissue infections clinic in San Francisco that also provided access to drug and alcohol counselling and treatment as well as social work support, dramatically reduced emergency department visits by 34% and surgical service admissions by 47%, saving over $US8,000,000 in the first year of operation [102]. A review of care provided to homeless patients with serious infections found improved clinical cure and retention in addiction care if infectious diseases and addiction consultation was provided alongside case management and OAT (odds ratio 3.15, *p* = 0.03 and odds ratio 5.46, *p* = 0.01, respectively) [77]. Furthermore, guidance needs to be available to medical staff so that clinicians are comfortable providing education and harm reduction when people who inject drugs require care. Clinician comfort was improved through a program that educated clinicians on identifying key moments of infection prevention in injecting drug use [103]. The provision of a brief skin and needle hygiene behavioural intervention in hospital to people who inject drugs reduced the rate of uncleaned skin injections (incidence rate ratio 0.34, 95% CI 0.20–0.59, *p* < 0.001) [104]. Further research is required to explore the efficacy and acceptability of these preventative strategies in the Australian context to decrease the current upward trend of these infections.

## AUTHOR CONTRIBUTIONS

Each author certifies that their contribution to this work meets the standards of the International Committee of Medical Journal Editors.

## CONFLICT OF INTEREST STATEMENT

Daniel O'Keefe has received investigator‐driven research funding from Gilead Sciences for work on hepatitis C unrelated to this manuscript. Peter Higgs has received investigator‐driven research funding from Gilead Sciences and AbbVie for work on hepatitis C unrelated to this manuscript. Joseph S. Doyle's institution has received investigator‐initiated research funding from Gilead Sciences and AbbVie and honoraria from Gilead Sciences and AbbVie. Andrew J. Stewardson's institution has received investigator‐initiated research funding from Merck, Sharp and Dohme. All other authors declare no competing interests.
